# Preliminary evidence for endoscopic surgery combined with postoperative anti-PD-1 immunotherapy in advanced recurrent nasopharyngeal carcinoma

**DOI:** 10.1186/s12885-023-11760-y

**Published:** 2023-12-21

**Authors:** Haoyuan Xu, Wanpeng Li, Huankang Zhang, Huan Wang, Li Hu, Yurong Gu, Dehui Wang

**Affiliations:** ENT institute and Department of Otorhinolaryngology, Eye & ENT Hospital, Fudan University, Shanghai, 200031 China

**Keywords:** Recurrent nasopharyngeal carcinoma, Anti-PD-1 immunotherapy, Endoscopic surgery, Propensity score matching, Survival

## Abstract

**Backgroud:**

Endoscopic surgery can be used as the main treatment for advanced recurrent nasopharyngeal carcinoma (rNPC). However, there is a huge clinical controversy about the need for consolidated immunotherapy after surgery.

**Methods:**

We performed a retrospective propensity score-matched analysis (1:2) of patients with locally advanced rNPC who underwent endoscopic nasopharyngectomy (ENPG) combined with anti-programmed cell death protein-1 (PD-1) monotherapy or ENPG alone. The survival rate was analyzed by Kaplan–Meier method. The primary endpoint was progression-free survival (PFS). The secondary endpoints included overall survival (OS), objective response rate (ORR) and disease control rate (DCR). Potential surgical-related complications and immune-related adverse events (AEs) were also assessed.

**Results:**

We recruited 10 patients receiving ENPG plus anti-PD-1 monotherapy and 20 receiving ENPG alone. During the mean follow-up of 23.8 months, a significant improvement in the 2-year PFS was detected in the consolidation immunotherapy group compared to the ENPG alone group (80.0% vs. 40.0%; HR = 0.258; 95% CI: 0.09–0.72; *p* = 0.04), while the 2-year OS in the consolidation immunotherapy group was not significantly longer than that in the ENPG alone group (90.0% vs. 75.0%; HR = 0.482; 95% CI: 0.08–3.00; *p* = 0.50). The incidence of surgical-related complications in the consolidation immunotherapy group and ENPG alone group was 70.0 and 60.0%, respectively. Immune-related AEs were similar between the toripalimab arm (75.0%) and the camrelizumab arm (66.7%). Surgical-related complications depend on symptomatic treatments. Immune-related AEs were mild and tolerable.

**Conclusions:**

Consolidation immunotherapy regimen for patients with advanced rNPC after ENPG compared to ENPG alone provides a superior PFS rate with a manageable safety profile.

**Supplementary Information:**

The online version contains supplementary material available at 10.1186/s12885-023-11760-y.

## Introduction

x`Nasopharyngeal carcinoma (NPC) is a common head and neck cancer with significant ethnic and regional heterogeneity, and is highly prevalent in East and South Asia, particularly in southern China [1]. The incidences of local recurrence in newly diagnosed NPC range from 10 to 20% after primary radical radiotherapy [2, 3]. At current, either endoscopic nasopharyngectomy or reirradiation is the standard recommended first-line treatment for recurrent nasopharyngeal carcinoma (rNPC) [4]. Reradiotherapy has achieved a relatively high local control rate and overall rate because intensity modulated radiation therapy (IMRT) enables radiation delivery to target tissue with precise positioning and some tumours are still sensitive to radiation [5, 6]. However, radiation resistance of residual lesions and irreversible adverse effects after radiotherapy limit its use. With the development of endoscopic transnasal surgery techniques and an in-depth understanding of the anatomical structure of the nasal skull base, endoscopic nasopharyngectomy (ENPG) can define the resection range, which significantly reduces the positive margin rate by 2–7% [7]. ENPG demonstrated a better survival outcome (5-year overall survival: 77.1% for ENPG vs. 55.5% for reirradiation), improved quality of life, and a significant decline of treatment-related complications for rNPC compared with reirradiation [8]. Our previous study suggested that the 3-year OS of patients treated with endoscopic surgery (59.3%) was significantly higher than that of patients treated with IMRT (34.7%, *p* < 0.001) in a cohort of 243 patients with advanced rNPC (rT3 and rT4) [9]. For these patients with rT3 and rT4 undergoing endoscopic surgery, there is still a risk of tumor residual and recurrence even if the surgical margins are negative. Therefore, there is significant untapped opportunity in further improving survival outcomes of these advancd rNPC patients.

Endemic nasopharyngeal carcinomas are characterized by high programmed death ligand-1 (PD-1) expression and intense infiltration of nonmalignant lymphocytes, which makes immunotherapy a promising treatment option in this setting [3, 10]. Immune checkpoint blockade therapies targeting the PD-1 receptor have shown significant treatment efficacy with a manageable safety profile for recurrent or metastatic nasopharyngeal carcinoma (r/mNPC) in several recently published prospective clinical trials, with an objective response rate of 20–34% [11–16]. Thus, we hypothesize that postoperative consolidation immunotherapy can further improve survival outcomes in patients with advanced rNPC after ENPG. In our study, we conducted a retrospective propensity score-matched study to compare outcomes of ENPG combined with postoperative anti-PD-1 monotherapy and ENPG alone in advanced rNPC patients.

## Methods

### Patient selection

**Fig. 1:**
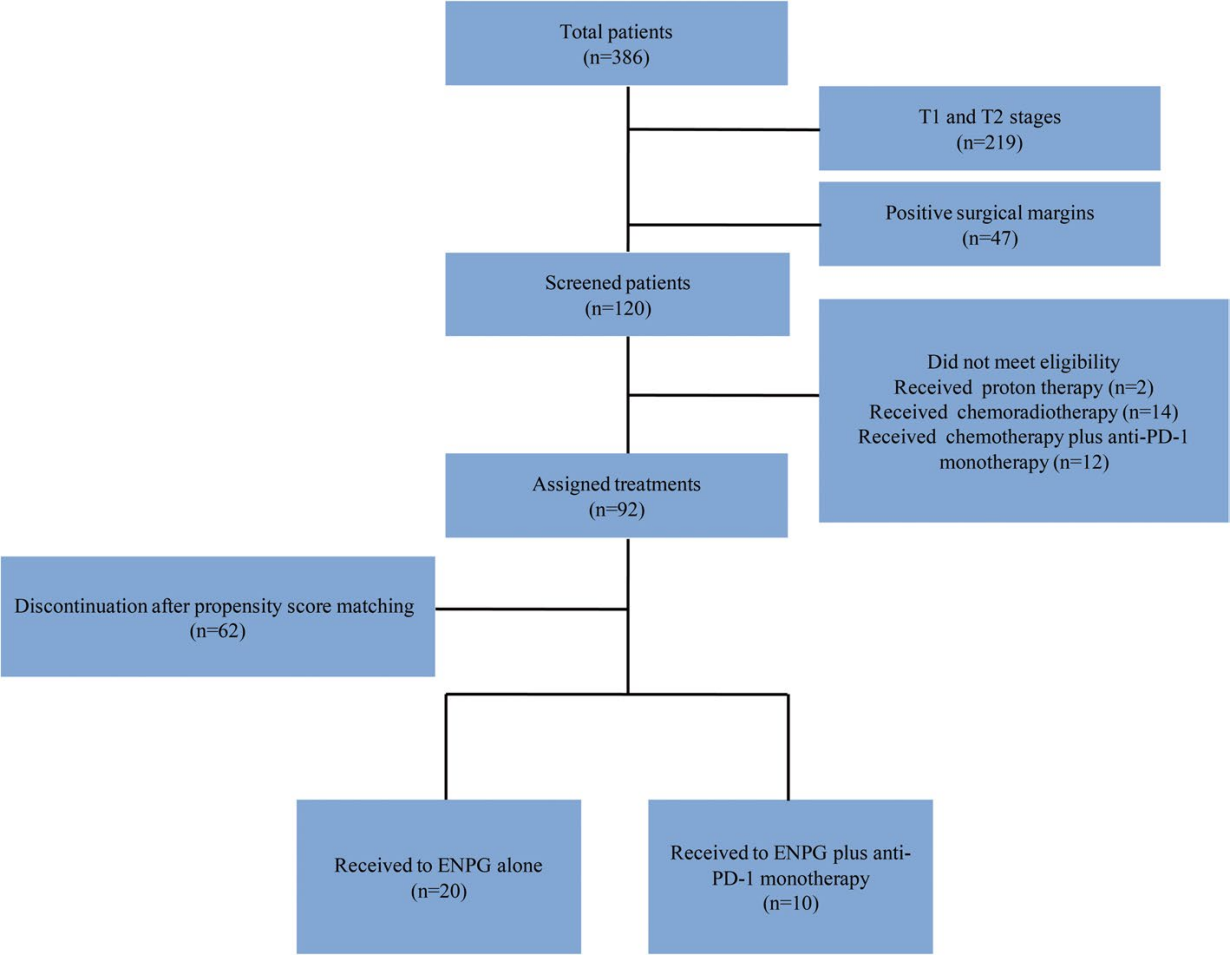
Selection criteria for the patients enrolled in the study. Propensity score matching was used to adjust for 10 related parameters: age, sex, body mass index, internal carotid artery embolization, pathological type, postoperative reconstruction, T stage, lymph node metastasis, period between recurrence and the last session of radiotherapy, and preoperative combined chemotherapy before surgery

The clinical data of patients who were diagnosed with locally advanced rNPC (rT3 and rT4) and underwent ENPG at the Department of Otorhinolaryngology of the Affiliated Eye, Ear, Nose, and Throat Hospital at Fudan University from April 2017 to March 2021 were reviewed. Patients were eligible for enrolment if they had pathologically confirmed tumour recurrence. The patients with T1 and T2 rNPC, or with positive surgical margins were all excluded. After the initial screening, 120 patients were enrolled for further evaluation. According to postoperative adjuvant therapy, 28 patients (2 received proton therapy, 14 received chemotherapy, 12 received chemotherapy plus anti-PD-1 monotherapy) were excluded. According to the demographic information and clinical characteristics of eligible cases, 10 relevant parameters (age, sex, body mass index, internal carotid artery embolization, pathological type, postoperative reconstruction, T stage, lymph node metastasis, period between recurrence and the last session of radiotherapy, and preoperative combined chemotherapy before surgery) were used for propensity score analysis. We created a relatively consistent cohort including 30 patients in total by matching two patients who did not receive any postoperative adjuvant therapy (defined as the ENPG alone group) with one patient who only received postoperative consolidation anti-PD-1 monotherapy (defined as the consolidation immunotherapy group) (Fig. 1). The current study was approved by the Institutional Review Board of the Affiliated Eye, Ear, Nose, and Throat Hospital at Fudan University.

### Surgical procedure

**Fig. 2:**
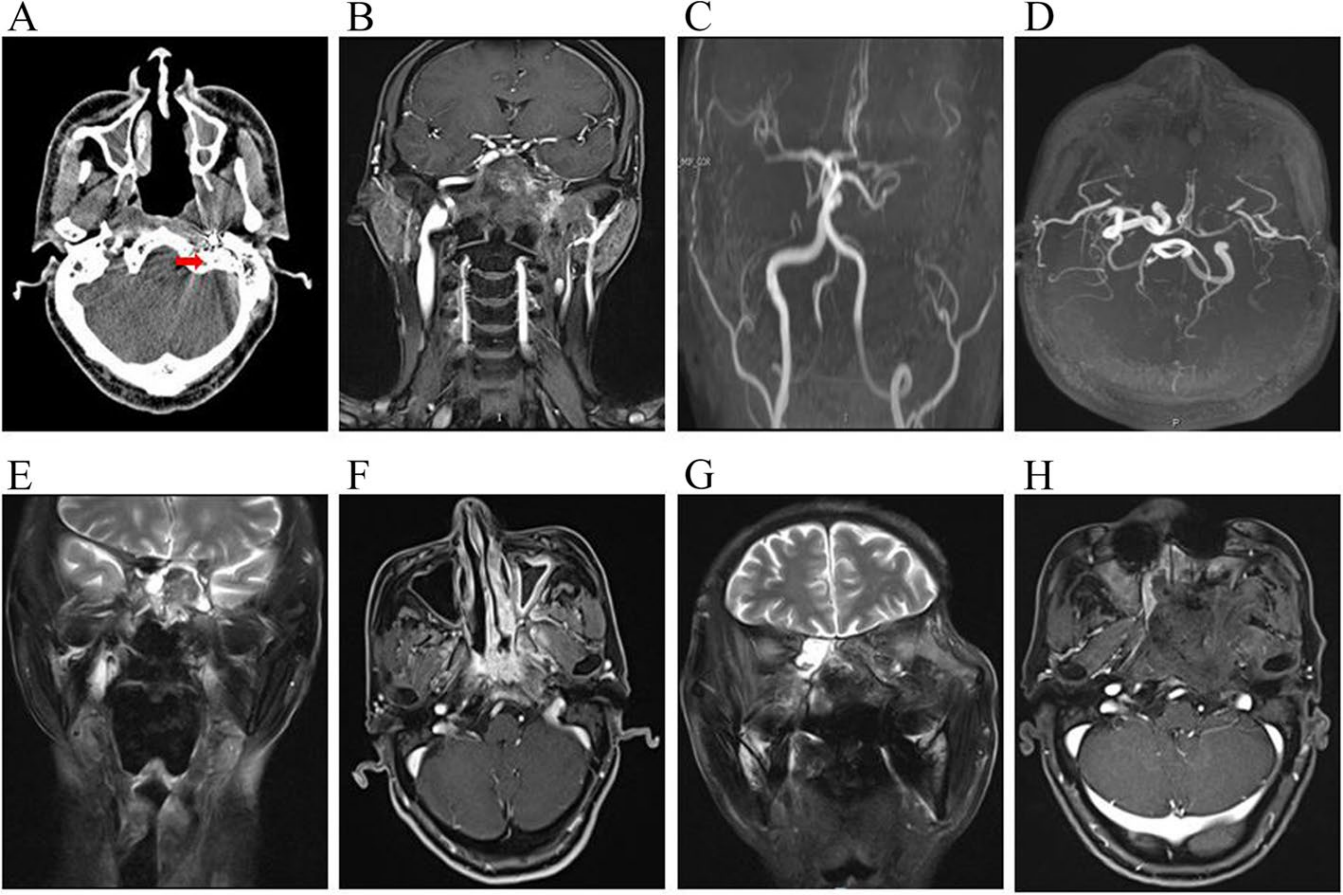
Imaging results of ICA embolization. **A** Horizontal enhanced CT demonstrates a diffuse soft tissue in the left nasopharyngeal lateral wall, parapharyngeal space and retropharyngeal area. Radial artifacts of high density martix coils were noted (arrow). **B** Coronal enhanced MRI demonstrates enhanced nodules on the left nasopharyngeal lateral wall involving the carotid sheath space and middle skull base bone. **C** and **D** MRA shows no development of the left ICA. The left middle cerebral artery is slightly thinner than the contralateral arttery, but the cerebral artery ring is generally intact. Enhanced MRI coronal T2 (**E**) and horizontal T1 (**F**) images showed soft tissue lesions on the posterior wall of the nasopharyngeal top and the left lateral wall, involving bilateral posterior pharyngeal head longus muscle, left choanal, pteryopalatine fossa, bilateral middle skull base bone and sphenoid sinus. The left internal carotid artery showed post-embolization changes. Enhanced MRI coronal T2 (**G**) and horizontal T1 (**H**) images demonstrated that the original lesions in the left nasopharynx and middle cranial fossa had been resected. CT: computed tomography; ICA: internal carotid artery; MRI: magnetic resonance imaging; MRA: magnetic resonance angiography

**Fig. 3:**
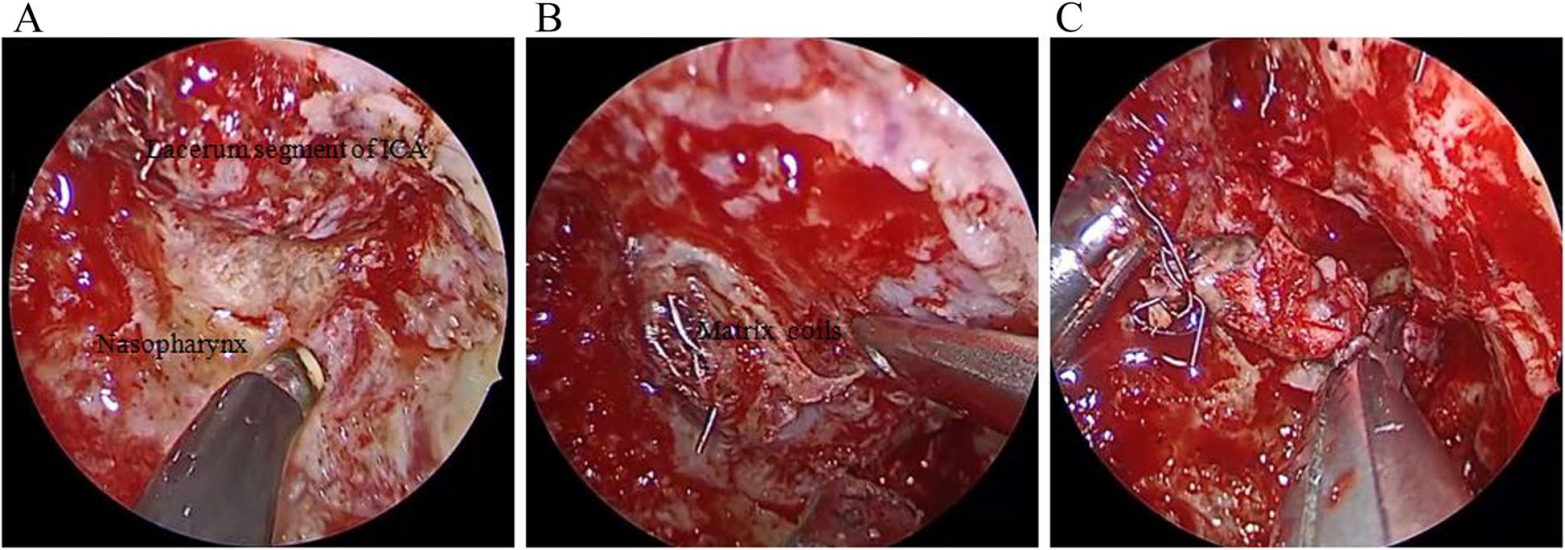
Excision of the ICA after embolization for rNPC. **A** The lacerum segment of the ICA was clearly exposed. **B** The lacerum segment of the ICA was separate, and the matrix coils were seen. (**C**) The ICA was safely removed after embolization. ICA: internal carotid artery; rNPC: recurrent nasopharyngeal carcinoma

**Fig. 4:**
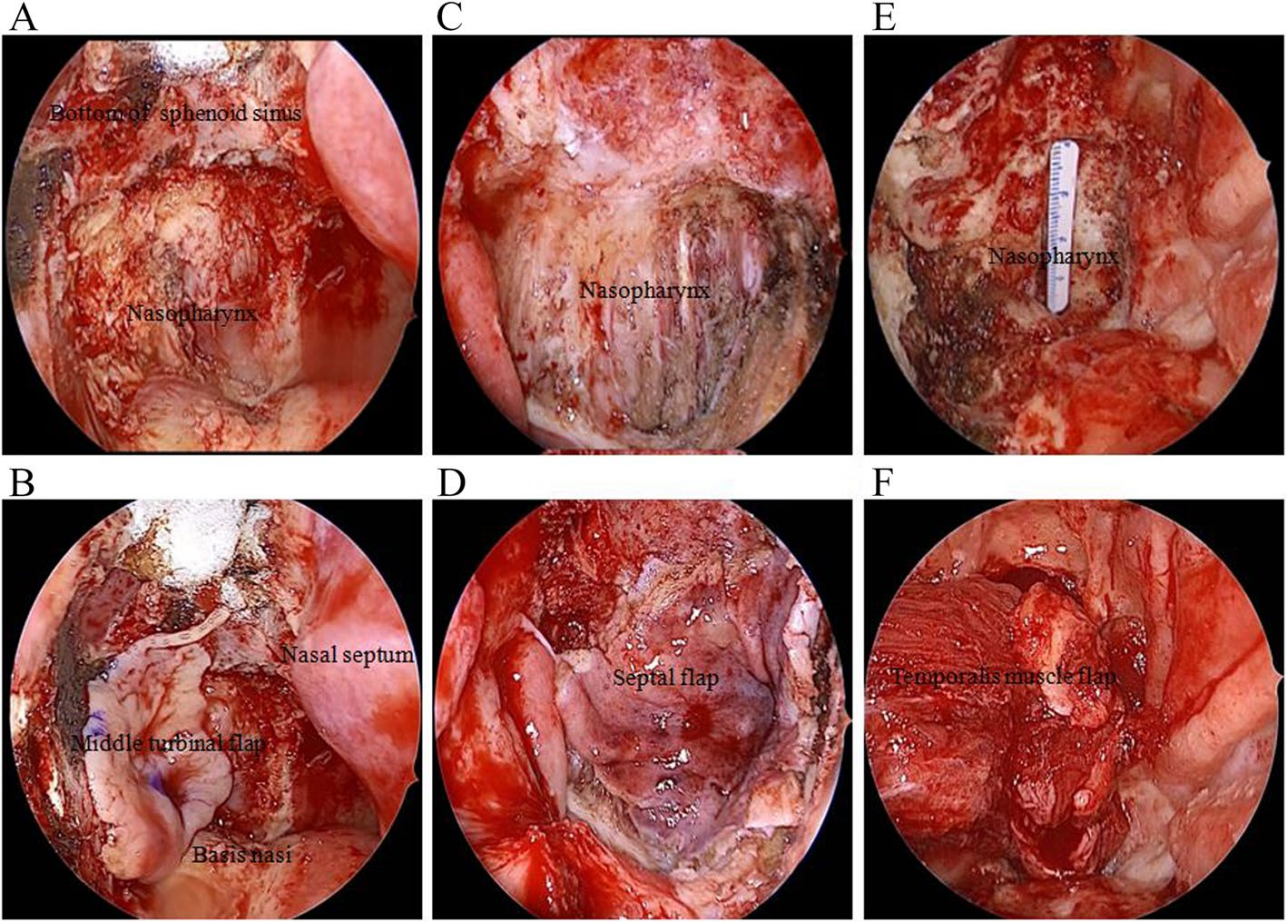
Reconstruction of skull base defect after salvage endoscopic nasopharyngectomy for locally advanced recurrent nasopharyngeal carcinoma. **A** and **B** Middle turbinal flap; **C** and **D** septum mucosa flap; and **E** and **F** temporal muscle flap

Based on the location and extent of the tumour, patient preferences, and consultations with the radiation oncologists and surgeons, a joint decision was made to perform salvage ENPG with different operative approaches. For patients with T3 and T4, we tend to believe that radical resection is feasible when the tumor invovles the skull base bone, pterygoid structures, paranasal sinuses, part of the dura mater and orbital structure, and other adjacent tissue. The detailed procedures in detail have been described in our previous articles [17, 18]. If the ballon occlusion test (BOT) result was negative, unilateral internal carotid artery (ICA) occlusion was performed immediately during operation (Figs. 2 and 3). If postoperative defect is large, a septal flap, temporalis muscle flap or dural substitute can be selected (Fig. 4). For patients with cervical lymph node metastasis, simultaneous or two-stage lymph node dissection was performed. The brief surgical procedure was as follows. An “L” incision was made in the lateral neck. The skin layer and platysma muscle were dissected to expose the sternocleidomastoid muscle. The internal jugular vein, accessory nerve, and sternocleidomastoid muscle were preserved. The range was from the mandibular margin down to the omohyoid muscle and from the sternocleidomastoid to the midline of the neck. Adipose tissue and lymph nodes were dissected layer by layer. All suspected enlarged lymph nodes, all layers of adipose tissue and involved glands in zones I-III were routinely removed. Suspicious lymph nodes in other regions were resected at the same time.

### Immunotherapy

The treatment was performed according to recommended protocols, the clinician’s suggestions, and the patient’s acceptance. Information on subsequent immunotherapy including start time, dosage, times of use, and end time was collected. All adverse events were defined according to the National Cancer Institute-Common Terminology Criteria for Adverse Events v.5.0.

### Assessment and outcomes

All patients were followed up and the last follow-up was completed in July 2023. Tumour response was evaluated by contrast-enhanced MRI and endoscopic examination by independent radiologists and clinicians until disease progression or death every 3 months. The primary endpoint was progression-free survival (PFS), defined as the time between admission and documented disease progression or death (any cause). Secondary endpoints were (1) objective response rate (ORR), defined as the proportion of patients with confirmed complete (CR) or partial response (PR); (2) disease control rate (DCR), defined as the proportion of patients with best response of CR, PR or stable disease (SD); and (3) overall survival (OS), defined as the time from admission until death (any cause) or censored at the last date of known survival.

### Statistical analysis

The intergroup differences in categorical variables were compared using the chi-square test. Descriptive parameters are summarized using simple descriptive statistics. The ORR and DCR were estimated on the full analysis set and the corresponding 95% CIs were calculated with the Clopper-Pearson method. OS and PFS were estimated using the Kaplan–Meier method. All tests were two-sided and a *p* value < 0.05 was considered significant. All statistical analyses including propensity score matching were conducted using the statistical software package SPSS version 25.0.

## Results

### Propensity matching

Supplementary Table 1 summarizes the patient demographics and clinical characteristics before propensity score matching. Before matching, there was a significant difference in ICA embolization (*p* = 0.001) and postoperative reconstruction approaches (*p* = 0.021) between the two groups, which may affect the results. We further performed rigorous propensity score matching to avoid an imbalanced covariate distribution. Supplementary Table 2 summarizes aspects consistent with the above after propensity score matching, showing that there was no significant disparity in the characteristics between the two groups, which reduced confounding factors that could influence outcomes. By forest plot for multivariable Cox regression analysis, we found that the results from propensity score matching and multivariable Cox regression are consistent. (Supplementary Fig. 1).

### Patient characteristics and oncological outcomes

**Fig. 5:**
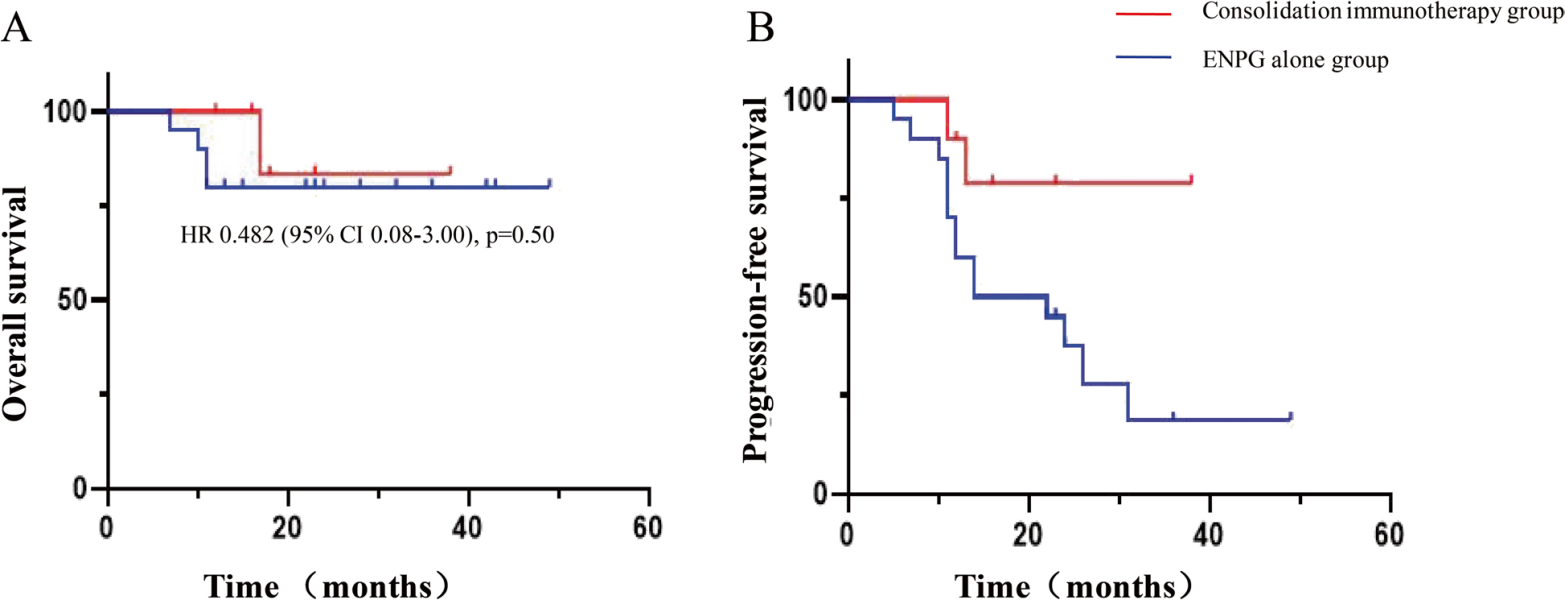
Kaplan-Meier analysis of (**A**) overall survival and (**B**) progression-free survival

A total of 30 patients were recruited for the final cohort (Table 1). The mean follow-up duration was 37.8 months (range: 7–66). During the study period, 46.7% (14 of 30) of patients relapsed (12 patients belonged to the ENPG alone group). The probability of death was 20.0% (6 of 30) (1 patient belonged to the consolidation immunotherapy group). Among the 6 patients who died, 3 died due to tumour progression, 1 died due to brain metastasis, 1 died due to lung metastasis, and 1 died due to massive internal carotid haemorrhage. The 1- and 2-year OS rates in the consolidation immunotherapy group were longer than those in the ENPG alone group (100.0% vs. 75.0, and 90.0% vs. 75.0%, respectively; hazard ratio (HR) = 0.482; 95% confidence interval (CI): 0.08–3.00; *p* value = 0.50) (Fig. 5a). In addition, the 1- and 2-year PFS rates in the consolidation immunotherapy group were significantly longer than those in the ENPG alone group (90.0% vs. 60.0, and 80.0% vs. 40.0%, respectively; hazard ratio (HR) = 0.258; 95% confidence interval (CI): 0.09–0.72; *p* value = 0.04) (Fig. 5b). Tumour responses at the last follow-up are summarized in Table 2. Seven (35.0% [95% CI 14.1–55.9]) patiens in the ENPG alone group and 6 (60.0% [95% CI 29.6–90.4]) patients in the consolidation immunotherapy group achieved an ORR (*p* = 0.19). Eight (40.0% [95% CI 18.5–61.5]) in the ENPG alone group and 8 (80.0% [95% CI 55.2–104.8]) patients in the consolidation immunotherapy group achieved a DCR (*p* = 0.03).

**Table 1:** Characteristics of enrolled patients

Clinical features	ENPG alone group (*n* = 20)	Consolidation immunotherapy group (*n* = 10)	N	%	*p*-value
Age, n (%)					1.000
≥50 years	12	6	18	60.0	
<50 years	8	4	12	40.0	
Sex, n (%)					1.000
Male	16	8	24	80.0	
Female	4	2	6	20.0	
Body mass index, n (%)					0.838
<18.5	2	1	3	10.0	
18.5–24.9	12	7	19	33.3	
>24.9	6	2	8	26.7	
ICA embolization, n (%)					1.000
Yes	18	9	27	90.0	
No	2	1	3	10.0	
Pathologic type, n (%)					1.000
WHO type II	12	6	18	60.0	
WHO type III	8	4	12	40.0	
Postoperative reconstruction, n (%)					0.677
Septal flap	7	2	9	30.0	
Middle turbinal flap	1	1	2	13.3	
Dural substitute	2	0	2	6.7	
Temporalis muscle flap	8	6	14	46.7	
No use	2	1	3	10.0	
T stage (initial status), n (%)					0.741
T2	3	2	5	16.7	
T3	16	8	24	80.0	
T4	1	0	1	3.3	
T stage (first relapse stauts), n (%)					1.000
T3	18	9	27	90.0	
T4	2	1	3	10.0	
Lymph node metastasis, n (%)					1.000
Yes	6	3	9	30.0	
No	14	7	21	70.0	
Period bewteen recurrence and the last session of radiotherapy, n (%)					1.000
≥3 years	12	6	18	60.0	
<3 years	8	4	12	40.0	
Preoperative combined chemotherapy before surgery, n (%)					0.958
Cisplatin	19	9	28	93.3	
Gemcitabine	12	6	18	60.0	
Docetaxel	7	3	10	33.3	
No	1	1	2	6.7	
Recurrence site previously irradiated, n (%)					0.301
Yes	18	10	28	93.3	
No	2	0	2	6.7	
Negative margin, n (%)					-
Yes	20	10	30	100.0	
No	0	0	0	0	

Abbreviation: *ENPG* endoscopic nasopharyngectomy, *ICA* internal carotid aetery, *WHO* World Health Organization

**Table 2:** Summary of tumor response

Variable	ENPG alone group (*n* = 20)	Consolidation immunotherapy group (*n* = 10)	*p*-value
Complete response, n (%)	3 (15.0)	1 (10.0)	0.704
Partial response, n (%)	4 (20.0)	5 (50.0)	0.091
Satable disaese, n (%)	1 (5.0)	2 (20.0)	0.197
Progressive disease, n (%)	12 (60.0)	2 (20.0)	0.038
Objective response % (95% CI)	35.0 (14.1–55.9)	60.0 (29.6–90.4)	0.193
Disease control rate % (95% CI)	40.0 (18.5–61.5)	80.0 (55.2–104.8)	0.038

Abbreviation: *ENPG* endoscopic nasopharyngectomy

### Surgical complications

During the perioperative period, none of the patients died due to the surgical complications. The most common complication was dysphagia (20.0%), followed by nasal congestion (13.3%), nasopharyngeal haemorrhage (10.0%), nasopharyngeal necrosis (6.7%), facial numbness (6.7%), limitation of mouth opening (6.7%), and cerebral infarction (3.3%). The chi-square test demonstrated that the rates of these complications were not significantly different bewteen the ENPG alone group and the consolidation immunotherapy group (*p* > 0.05) (Table 3).

**Table 3:** Common surgery-related complications

Complicaions	ENPG alone group No./Total (%)	Consolidation immunotherapy group No./Total (%)	χ2	*p*-value
Nasal congestion	2 (10.0)	2 (20.0)	0.577	0.448
Dysphagia	5 (25.0)	1 (10.0.)	0.938	0.333
Nasopharyngeal hemorrhage	2 (10.0)	1 (10.0)	0	1.000
Nasopharyngeal necrosis	2 (10.0)	0 (0)	1.071	0.301
Facial numbness	1 (5.0)	1 (10.0)	0.268	0.605
Limitation of mouth opening	1 (5.0)	1 (10.0)	0.268	0.605
Cerebral infraction	1 (5.0)	0 (0)	0.517	0.472

Abbreviation: *ENPG* endoscopic nasopharyngectomy

### Immunotherapy and related adverse events

The course of immunotherapy was reviewed, and all patients in the consolidation immunotherapy group began PD-1 monotherapy (4 in toripalimab and 6 in camrelizumab) within 2 to 6 weeks postoperatively. Patients received toripalimab 3 mg/kg or camrelizumab 200 mg once every 3 weeks via intravenous infusion. Except for patients with disease progression, others were still in the consolidation stage after the last follow-up period. Ninety percent (9 of 10) completed treatment for at least 1 year, and 20% (2 of 10) even completed treatment for at least 2 years. Two patients achieved CR (2 in camrelizumab) and 4 achieved PR (3 in toripalimab and 1 in camrelizumab). The most common immune-related complications were rash (*n* = 2) and nausea (n = 2), and none of the patients discontinued treatment due to intolerable adverse events (Table 4).

**Table 4:** Common immune-related adverse events

Complications	Toripalimab (*n* = 4)	Camrelizumab (*n* = 6)
Rash	1	1
Asthenia	0	1
Nausea	1	1
Pruritus	0	1
Hypothyroidism	1	0

### Treatment after tumor progression

**Fig. 6:**
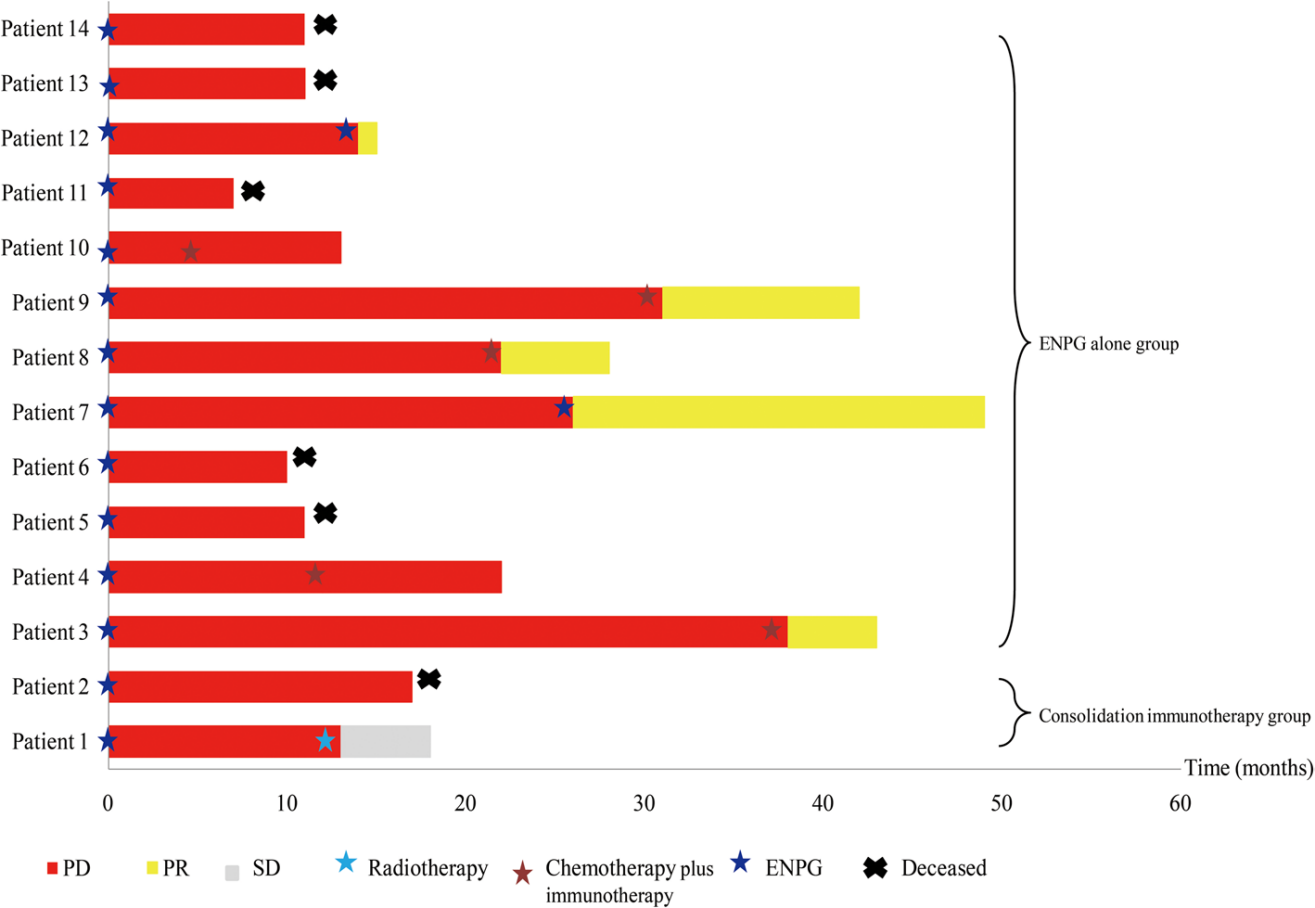
Swimming plot demonstrates the subsequent treatment process for patients in the progressive stage

In addition to the 6 died patients, we obtained subsequent treatment information for another 8 patients with secondary tumour progression (Fig. 6). Among the 7 cases of relapse in the ENPG alone group, 2 underwent secondary salvage ENPG and 5 received gemcitabine plus platinum (GP) combined with immunotherapy. Five patients achieved a temporary partial response while 2 patients did not respond to the GP combined with immunotherapy regimen and were in the disease progression stage. One recurrent patient in the consolidation immunotherapy group achieved stable disease by adding radiotherapy.

## Discussion

Local recurrence in NPC remains a difficult topic, presenting many challenges in management. The majority of patients who fail the first local treatment have only local recurrence without distant metastases [19]. Thus, the aim of offering salvage treatment to these patients is to achieve control of local disease to have a chance of cure, as well as alleviate any current and potential symptoms. The most common treatment for rNPC is salvage surgery and IMRT. Evidence is accumulating that endoscopic surgery is superior to IMRT in the treatment of rNPC [9, 20]. Hua et al. reported that the 3-year overall survival (OS) in surgery groups of rT3 and rT4 tumours was 68.8 and 36.9%, respectively, which was longer than that of patients receiving IMRT [5]. Several studies have suggested that recurrences amenable to surgery with favourable outcomes and a high likelihood of negative margins include the majority of rT1 and early rT2 disease and selected rT3 disease with a small disease volume and minimal skull base involvement [21–23]. With the development of arterial embolization techniques and the accumulation of surgeons’ anatomical experience related to the nasopharyx and skull base, indications are expanding to include rT3 and rT4 disease [9, 24–26]. Wong et al. [25] reported favourable patient outcomes with advanced rNPC (rT3 and rT4) who underwent an endoscopic approach, and the two-year OS (66.7%) and DFS (40.0%) results basically agreed with the our previous data (OS: 67.1%; DFS: 37.3%) [27]. However, the two-year OS (75.0%) and DFS (40.0%) in the ENPG group of this study were slightly higher, which may be interpreted as a highly selective cohort, in which most patients received ICA embolization and had negative surgical margins without any postoperative adjuvant therapy. In fact, there is a lack of two similar cohorts with which to compare the survival outcomes because of multiple confounding factors.

In our study, the removal of the advanced tumour resulted in a large wound, and the assured negative surgical margins may not exclude the scattered tumour residual, thus becoming the main cause of recurrence. Therefore, postoperative adjuvant therapy may contribute to the reduced recurrence rate for these patient poulations. Increasing studies have suggested that NPC is associated with the development of PD-1/L1 antibodies and is the subject of several ongoing pivotal phase III studies comparing PD-1 antibodies in combination with chemotherapy or radiation therapy (RT), as well as that it could be practice-changing in the palliative or radical management of NPC if the results are positive. A prospective study reported that the addition of anti-PD-1 blockers to GP chemotherapy provided superior survival parameters than GP alone while maintaining a manageable safety profile for recurrent or metastatic rNPC [14]. In addition, several studies have shown promising activities of anti-PD-1 monotherapy for chemorefractory NPCs [12]. However, the role of anti-PD-1 blockade combined with ENPG for rNPC as adjunctive therapy has not been evaluated. To date, this is the first case-matched study to show that ENPG combined with postoperative consolidation anti-PD-1 monotherapy improved the PFS, ORR, DCR, and OS of patients with advanced rNPC compared with ENPG alone. A recent study reported that 9 patients with advanced rNPC who had not responded to immunotherapy received salvage skull base surgery. The 2-year OS and PFS rates were 25.0 and 29.2%, respectively [28], which are significantly lower than those in the immunotherapy group of our study (OS: 90.0%; DFS: 80.0%). This finding suggests that patients with advanced rNPC who receive postoperative consolidation immunotherapy may achieve better survival parameters than those who receive salvage ENPG after immunotherapy failure.

Here, similar surgery-related complications occurred in both groups. These complications were mainly caused by extensive excision, including important nerves and blood vessel structures. Improvement of complications depends on symptomatic treatments, but complete recovery is difficult. The use of antibiotics, nasal irrigation, nasal spray of glucocorticoid and mucolytic expectorants can partially relieve these symptoms in approximately 4 weeks. Failure to establish collateral circulation after ICA embolization was the main cause of cerebral infarction [29]. Nasopharyngeal necrosis leads to unbearable headaches and even ICA involvement, and, thus requires subsequent debridement.

Although consolidation immunotherapy significantly optimized patient survival parameters, there is a high risk of drug-related adverse events. Previous studies have shown that all patients in the toripalimb arm and 84% of patients in the camrelizumab arm were reported to have immune-related adverse events [14, 27], which comprised all adverse events (3 of 4 in toripalimb and 4 of 6 in camrelizumab) in our data. Capillary haemangioma found as a unique toxicity developed in 88% of camrelizumab subjects [13], but was not observed in our study. Additionally, there were no severe adverse events that led to discontinuation of consolidation immunotherapy.

There are several other limitations of the study. First, the number of cases included in the two groups was relatively small after propensity matching and the duration of follow-up was relatively short. Amplifying the sample size and extending the follow-up time contribute to evaluating the efficacy and safety of postoperative consolidation immunotherapy. Next, plasma Epstein-Barr virus (EBV) DNA titers that could be used for monitoring disease progression were not evaluated in this study, even though we eliminated multiple confounders that could influence outcomes. Last, the level of evidence from retrospective studies is relatively limited. Our team is conducting a prospective randomized controlled trial in patients with rNPC treated with or without tirelizumab after ENPG (Ethics Registration Number: 2021079).

## Conclusions

In summary, consolidation immunotherapy regimen for patients with advanced rNPC after ENPG compared to ENPG alone provides a superior PFS rate with a manageable safety profile. A large and prospective study would facilitate this conclusion.

### Supplementary Information

**Additional file 1: Supplementary Table 1. **Patient information before propensity score matching. **Supplementary Table 2.** Patient information after propensity score matching. **Supplementary Figure 1.** A forest plot of multivariate Cox analysis in the propensity score matching cohort.

## Data Availability

The raw data supporting the conclusions of this article will be made available by the authors. Further inquiries can be directed to the corresponding authors.
