# Supplementary material for: Preliminary evidence for endoscopic surgery combined with postoperative anti-PD-1 immunotherapy in advanced recurrent nasopharyngeal carcinoma
Source: BMC Cancer. 2023 Dec 21;23:1259. doi: 10.1186/s12885-023-11760-y (PMC10734134; doi:10.1186/s12885-023-11760-y)
Supplement: Supplementary file 1 — Additional file 1: Supplementary Table 1. Patient information before propensity score matching. Supplementary Table 2. Patient information after propensity score matching. Supplementary Figure 1. A forest plot of multivariate Cox analysis in the propensity score matching cohort. [file 12885_2023_11760_MOESM1_ESM.docx]

Supplementary Table 1. Patient information before propensity score matching

| Clinical features | ENPG alone group (n=82) | Consolidation immunotherapy group (n=10) | χ2 | p-value |
| --- | --- | --- | --- | --- |
| Age, n (%)  ≥50 years  ＜50 years | 53 (64.6)  29 (35.4) | 6 (60.0)  4 (40.0) | 0.084 | 0.773 |
| Sex, n (%) |  |  | 0.020 | 0.888 |
| Male  Female | 64 (78.0)  18 (22.0) | 8 (80.0)  2 (20.0) |  |  |
| Body mass index, n (%) |  |  | 0.256 | 0.880 |
| ＜18.5  18.5-24.9  ＞24.9 | 13 (15.9)  55 (67.1)  14 (17.0) | 1 (10.0)  7 (70.0)  2 (20.0) |  |  |
| ICA embolization, n (%) |  |  | 10.970 | 0.001* |
| Yes  No | 29 (35.4)  53 (64.6) | 9 (90.0)  1 (10.0) |  |  |
| Pathologic type, n (%) |  |  | 0.055 | 0.814 |
| WHO type II  WHO type III | 46 (56.1)  36 (43.9) | 6 (60.0)  4 (40.0) |  |  |
| Postoperative reconstruction, n (%) |  |  | 11.590 | 0.021* |
| Septal flap | 36 (43.9) | 2 (20.0) |  |  |
| Middle turbinal flap | 5 (6.1) | 1 (10.0) |  |  |
| Dural substitute | 6 (7.3) | 0 (0) |  |  |
| Temporalis muscle flap | 13 (15.9) | 6 (60.0) |  |  |
| No use | 22 (26.8) | 1 (10.0) |  |  |
| T stage, n (%) |  |  | 1.506 | 0.220 |
| T3  T4 | 59 (72.0)  23 (28.0) | 9 (90.0)  1 (10.0) |  |  |
| Lymph node metastasis, n (%) |  |  | 0.150 | 0.699 |
| Yes  No  Period bewteen recurrence and the last session of radiotherapy, n (%)  ≥3 years  ＜3 years  Preoperative combined chemotherapy before surgery, n (%)  Yes  No | 20 (24.4)  62 (75.6)  51 (62.2)  31 (37.8)  63 (76.8)  19 (23.2) | 3 (30.0)  7 (70.0)  6 (60.0)  4 (40.0)  9 (90.0)  1 (10.0) | 0.018    0.909 | 0.893      0.340 |

Abbreviation: ENPG, endoscopic nasopharyngectomy; ICA: .internal carotid aetery; WHO, World Health Organization. *p value＜0.05 was considered statistically significant.

Supplementary Table 2. Patient information after propensity score matching

| Clinical features | ENPG alone group (n=20) | Consolidation immunotherapy group (n=10) | χ2 | p-value |
| --- | --- | --- | --- | --- |
| Age, n (%)  ≥50 years  ＜50 years | 12 (60.0)  8 (40.0) | 6 (60.0)  4 (40.0) | 0 | 1.000 |
| Sex, n (%) |  |  | 0 | 1.000 |
| Male  Female | 16 (80.0)  4 (20.0) | 8 (80.0)  2 (20.0) |  |  |
| Body mass index, n (%) |  |  | 0.355 | 0.838 |
| ＜18.5  18.5-24.9  ＞24.9 | 2 (10.0)  12 (60.0)  6 (30.0) | 1 (10.0)  7 (70.0)  2 (20.0) |  |  |
| ICA embolization, n (%) |  |  | 0 | 1.000 |
| Yes  No | 18 (90.0)  2 (10.0) | 9 (90.0)  1 (10.0) |  |  |
| Pathologic type, n (%) |  |  | 0 | 1.000 |
| WHO type II  WHO type III | 12 (60.0)  8 (40.0) | 6 (60.0)  4 (40.0) |  |  |
| Postoperative reconstruction, n (%) |  |  | 2.321 | 0.677 |
| Septal flap | 7 (35.0) | 2 (20.0) |  |  |
| Middle turbinal flap | 1 (5.0) | 1 (10.0) |  |  |
| Dural substitute | 2 (10.0) | 0 (0) |  |  |
| Temporalis muscle flap | 8 (40.0) | 6 (60.0) |  |  |
| No use | 2 (10.0) | 1 (10.0) |  |  |
| T stage, n (%) |  |  | 0 | 1.000 |
| T3  T4 | 18 (90.0)  2 (10.0) | 9 (90.0)  1 (10.0) |  |  |
| Lymph node metastasis, n (%) |  |  | 0 | 1.000 |
| Yes  No  Period bewteen recurrence and the last session of radiotherapy, n (%)  ≥3 years  ＜3 years  Preoperative combined chemotherapy before surgery, n (%)  Yes  No | 6 (30.0)  14 (70.0)  12 (60.0)  8 (40.0)  19 (95.0)  1 (5.0) | 3 (30.0)  7 (70.0)  6 (60.0)  4 (40.0)  9 (90.0)  1 (10.0) | 0  0.268 | 1.000  0.605 |

Abbreviation: ENPG, endoscopic nasopharyngectomy; ICA: .internal carotid aetery; WHO, World Health Organization.


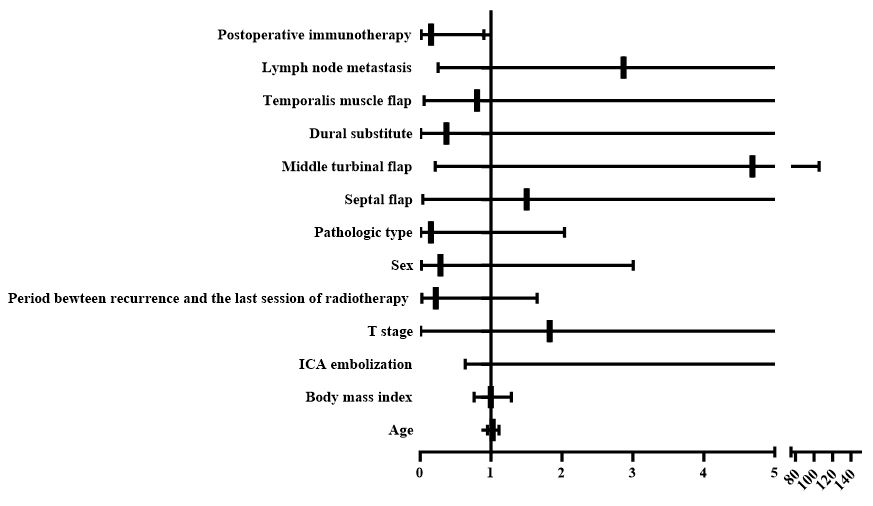


Supplementary Figure 1: A forest plot of multivariate Cox analysis in the propensity score matching cohort.
